# Intentional injury reported by young people in the Federated States of Micronesia, Kingdom of Tonga and Vanuatu

**DOI:** 10.1186/1471-2458-8-145

**Published:** 2008-04-30

**Authors:** Ben J Smith, Philayrath Phongsavan, Dale Bampton, Genevieve Peacocke, Mercedes Gilmete, Drew Havea, Tien Chey, Adrian E Bauman

**Affiliations:** 1 Department of Health Science, Monash University, PO Box 527 Frankston, Victoria, 3199, Australia; 2 School of Public Health, Lev 2, Medical Foundation Blg K25, University of Sydney, New South Wales, 2006, Australia; 3 Maurice Blackburn Cashman Pty Ltd, Level 20, 201 Elizabeth Street, Sydney, NSW, 2000, Australia; 4 Pfizer Australia, PO Box 57, West Ryde, New South Wales, 2114, Australia; 5 PO Box 723, Kolonia, Pohnpei, 96941, Federated States of Micronesia; 6 Training Group of the Pacific, PO Box 132, Nuku'alofa, Tonga

## Abstract

**Background:**

Intentional injury presents a threat to the physical and psychological well being of young people, especially in developing countries, which carry the greatest part of the global injury burden. While the importance of this problem is recognized, there are limited population data in low and middle income countries that can guide public health action. The present study investigates the prevalence and distribution of intentional injury among young people in three Pacific Island societies, and examines behavioural and psychosocial factors related to risk of intentional injury.

**Methods:**

Population surveys were conducted with **s**tudents aged 11–17 years in Pohnpei State in the Federated States of Micronesia (n = 1495), the Kingdom of Tonga (n = 2808) and Vanuatu (n = 4474). Surveys measured self-reported injury and intentional injury, sources of intentional injury, and the range of behavioural, psychological, educational and social variables that may be related to injury risk.

**Results:**

Among boys and girls aged 14–17 years the respective period prevalence of intentional injury was 62% and 56% in Pohnpei, 58% and 41% in Tonga, and 33% and 24% in Vanuatu. The prevalence of intentional injury declined with age in Tonga and Vanuatu, but there was little evidence of an age-trend in Pohnpei. Across the three societies, the major sources of intentional injury among boys were 'other persons' followed by boyfriends/girlfriends and fathers. Mothers, boyfriends/girlfriends and other persons were primary sources of injury among girls. An intentional injury was reported more often by those who had been bullied (OR 1.40–1.66, P < 0.05), by regular smokers in Tonga and Vanuatu (OR 1.52–2.21, P < 0.05), and illicit drug users in Pohnpei and Vanuatu (OR 1.87–1.92, P < 0.05).

**Conclusion:**

Intentional injury was reported extensively in these three populations. Interventions directed towards the school environment and which take into account the role of bullying and drug use need to be considered.

## Background

Global data on the burden of injury show that in 2002 intentional injuries accounted for 1.5% of Disability Adjusted Life Years (DALYs) among 5–14 year old girls and 2% among boys in this age group [1]. Among adolescents and young adults (15–29 years) the contribution of intentional injury to total DALYs was slightly lower in females (1.2%), but dramatically higher in males (8.9%) [1]. While mortality represents a significant component of this impact, it is estimated that there are 20–40 victims of non-fatal violence for every death [2].

All forms of injury present threats to physical and psychological well-being, and for children and adolescents an important focus of research has been the nature and impact of injury arising from interpersonal violence. This encompasses violence inflicted by parents and family members, peers, dating partners, teachers and other authority figures such as police. The 1997–98 Health Behavior in School Children (HBSC) survey of 11–15 year old students in Europe and North America found that fighting caused about 4% of total injury [3]. Additional measures concerning violent behaviours used in five of the HBSC countries revealed that between 11.2% and 17.9% of students suffered at least one injury due to fighting in the past 12 mths [4]. Research among younger aged students (school years 4–6) in China found that 23% had experienced violence at a level likely to cause injury in the family context, while 14% had been exposed to this from peers and 4% from teachers [5]. Surveys among students of the same age in Korea found higher levels of exposure to violence in the family (51%) or from teachers (44%), but a similar prevalence from peers (14%) [5].

Exposure to harsh discipline from parents and authority figures has been investigated in a number of countries. In Egypt it has been reported that 37% of preparatory and high school students had been disciplined physically by parents and 26% had been physically injured as a result of such discipline [6]. In the same study, about 70% reported being physically disciplined by teachers, with just under one-quarter reporting injury as a result [7]. The WorldSafe study, a cross-national comparison of the prevalence of different methods of moderate and severe punishments by mothers, revealed that there were at least three countries (Egypt, India and Philippines) where more than 20% of mothers reported striking their child with an object or in an area other than buttocks in the past 6 months [8].

The psychological and behavioural impacts of interpersonal violence, which include depression, anxiety, substance abuse, delinquent behaviours, poor school performance and self-harm [2], mean that this continues to be a public health priority. There is a particular need for population-based data in low and middle-income countries, which account for about 95% of the global burden of injury [9]. Many of these countries are experiencing social and cultural upheavals related to poverty and urbanisation, and in some locations violence has become the number one mechanism of injury death [10].

The purpose of the present study is to examine the extent and sources of intentional injury among young people in three Pacific island societies; the state of Pohnpei in the Federated States of Micronesia (FSM), Vanuatu and the Kingdom of Tonga. Injury and violence have been recognized as a health priority across the Pacific region, but there are little country-specific data to guide action. The health and education authorities in these locations agreed to collaborate with UNICEF in the development of a Lifeskills program for adolescents, and the data presented here formed part of the baseline measures for this initiative. This investigation provides an opportunity to examine the extent of intentional injury in populations with high proportions of children and adolescents [11] which are experiencing challenges related to globalization, including economic changes and greater interaction between traditional values and western behavioural mores [12,13]. Comparison of the behavioural and psychosocial factors associated with intentional injury across the societies assists in the identification of common issues to address in policy and programs, as well as recognizing those which are distinct to each location.

## Methods

### Sampling methods and study population

The Health Behaviour and Lifestyle of Pacific Youth (HBLPY) study comprised cross-sectional surveys of representative samples of school students aged 11 to 17 years in Tonga, Vanuatu and Pohnpei State of FSM. The methodology of this study has been described in detail elsewhere [14].

Briefly, cluster random sampling was used to select primary and secondary school students; international schools and schools located in remote areas were removed from the sampling frame. Cluster sampling is used in the HBSC studies in numerous countries [4] because of the logistical difficulties of recruiting a simple random sample of students across all schools in a population. In FSM, surveys were conducted in Pohnpei state only because of its accessibility and the interest of stakeholders in collecting adolescent health information. Given the social and developmental diversity of the states of FSM, the findings can therefore only be generalised to students in Pohnpei. The island groups of Tongatapu, Vava'u and Hapa'i were included in Tonga. The proportion of schools participating in each country ranged from all secondary schools in Pohnpei (7 schools), to 75% of eligible secondary schools in Vanuatu (32 schools), and 20% (29) and 43% (20) of eligible primary and secondary schools in Tonga, respectively. All schools invited to participate in the study agreed to do so. Primary school students were not included in Pohnpei and Vanuatu because of their variable literacy levels. As a result, there was some variation in the age range of participating students in each location: 14–17 years in Pohnpei, 11–17 years in Tonga, and 12–17 years in Vanuatu.

### The questionnaire

Students in all locations were asked about: socio-demographic characteristics; substance use (smoking, alcohol, illicit drug use); mental well-being; perceptions of the school environment; ease of communication with family, peers and others; bullying, and; injury. The injury and violence questions asked students to report the frequency of physical injury during the last 12 months. Following this students were asked if they had experienced a physical injury that was deliberately caused by: father; mother; teacher; police; boyfriend/girlfriend, or; other persons.

The questionnaire was extensively pre-tested with school students and key stakeholders to ensure relevance, comprehension and acceptability. All surveys were administered in the local languages.

### Data collection

The data collection protocol for this study was ethically reviewed and approved by UNICEF and the Ministries of Education, Health, and Youth Affairs in each country. Permission for implementation of the survey was also obtained directly from the relevant school systems. Information about the survey was distributed to parents and only those providing notification of a wish not to take part were excluded (passive consent). Consenting students were informed of the aims and procedures of the study, their right to abstain from completing any of the questions or withdraw from the survey, and the confidentiality of all data collected. Students self-completed the questionnaire either in their classrooms or in designated areas under the supervision of survey staff. To ensure students' privacy and to allow for anonymous participation, teachers or any authority figures were not present during the surveys. The surveys were conducted between September and October 2000 in Vanuatu, October and November 2000 in Tonga, and in April 2001 in Pohnpei.

### Statistical analysis

Analyses were conducted using SAS software V9.1.3 (SAS Institute Inc., Cary, NC, USA, 2002–2003). We used *Surveymeans *and *Surveylogistic *procedures to account for the clustering within schools. Descriptive statistics on the source of intentional injury were tabulated by sex and age. As indicated above and described elsewhere [14], the sampling fractions were high in each population, hence finite population correction factors of 0.935, 0.904 and 0.791 were used for Tonga, Vanuatu and Pohnpei respectively. All 95% confidence intervals accounted for design effects and were corrected for finite population sampling.

Independent factors associated with intentional injury were investigated using stepwise multiple logistic regression analyses. Factors of interest were substance use (weekly smoking, illicit drug use), behavioural factors (bullying others, being bullied, communication with friends/family), school environment and teacher ratings. Different models were constructed for each country; age and sex were included in all models and other variables were entered if p < 0.20 and retained if p < 0.15 using the SAS *Proc Logistic *function. Highly collinear variables (Spearman correlation >0.4) were identified by examining the correlation matrix of all the independent variables. Only one correlation between "school score" and "teacher score" met this threshold. The change in deviance (as a chi-square) when each of these variables was added or subtracted from a model was used to select the variable that was excluded.

## Results

### Respondent characteristics

The number of participating students in Pohnpei was 1495, while in Tonga the sample size was 2808 and in Vanuatu it was 4474. The response rates achieved in each location were 80%, 62% and 75%, respectively. Within each population there were similar proportions of boys and girls at each age level.

### Age and sex trends in intentional injury

**Figure 1:**
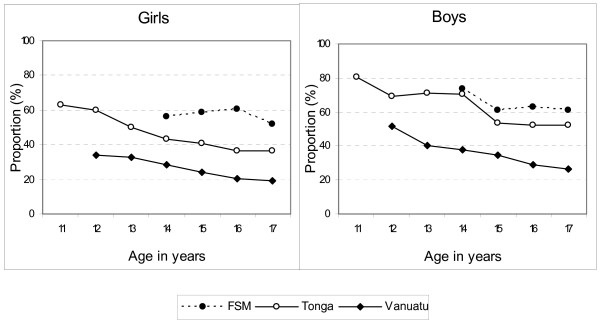
Prevalence of intentional injuries from any source in past 12 months by age and sex.

In Tonga and Vanuatu there were declines in the proportion of boys and girls reporting an intentional injury between the youngest and oldest age groups, with boys more often reporting an intentional injury at each age level (Figure 1). In Pohnpei there was a slightly lower period prevalence of intentional injury at the oldest age level among girls, compared with the 14–16 years age levels. Among boys, 14 year olds most often reported an intentional injury and the level remained reasonably stable from age 15 years onwards. In Pohnpei intentional injury was more often reported by boys than girls at age 14, with only small difference between the sexes at the other age levels. Vanuatu showed the lowest levels of intentional injury across the three populations.

### Sources of intentional injury

Table 1 shows that there was wide variation in the period prevalence of intentional injuries from any source among 14–17 year olds, from 33% in Vanuatu to 62% in Pohnpei among boys, and 24% in Vanuatu to 56% in Pohnpei among girls. In Pohnpei there was not a significant difference in the proportion of boys and girls reporting an intentional injury, whereas in the other societies boys most often reported an intentional injury from at least one source.

**Table 1:** Sources of intentional injury in past 12 months among adolescents aged 14–17 years

Variables	Boys: period prevalence % (95% CI)		
	
	Pohnpei n = 712	Tonga n = 502	Vanuatu n = 1576
Father	26 (18–35)	15 (12–18)	10 (8 -11)
Mother	23 (15–31)	12 (7 -16)	6 (5 -8)
Teacher(s)	13 (9–16)	14 (11–17)	7 (5 -9)
Police	13 (10–15)	11 (8 -14)	5 (3 -6)
Boyfriend/Girlfriend	31 (25–37)	27 (22–32)	12 (10–13)
Other person	38 (35–41)	41 (36–46)	21 (18–24)
Any above	62 (53–72)	58 (52–65)	33 (30–36)
	Girls: period prevalence % (95% CI)		
	
	Pohnpei n = 763	Tonga n = 668	Vanuatu n = 1478
	
Father	24 (21–26)	10 (7 -14)	10 (8 -11)
Mother	39 (33–44)	11 (9 -14)	10 (7 -12)
Teacher(s)	7 (6 -8)	10 (7 -12)	5 (3 -7)
Police	4 (3 -4)	4 (3 -6)	2 (2 -3)
Boyfriend/Girlfriend	24 (22–27)	18 (15–22)	8 (6 -10)
Other person	22 (20–23)	24 (20–28)	10 (9 -12)
Any above	56 (52–60)	41 (35–46)	24 (21–27)

n = number with non-missing value of any of the category.

95% CI adjusted for clustering effect by school and corrected for finite population sampling.

Among boys the most frequently cited source of injury was "other persons", although in Pohnpei the period prevalence of injury from this source did not differ significantly from that of the next two primary sources, namely boyfriend/girlfriend and fathers. In Tonga boyfriend/girlfriend ranked as the second major source of injury, followed by fathers, mothers, teachers and police, which were reported with similar levels of frequency. Boyfriends/girlfriends and fathers were the second most frequently reported sources of injury in Vanuatu, followed by the other sources.

The sources of intentional injury reported by girls showed some differences from that of boys. In Pohnpei mothers were the most frequently reported source, followed by fathers, boyfriends/girlfriends and other persons. In Tonga, other persons and boyfriends/girlfriends were most often identified, as for boys, followed by fathers, mothers and teachers. In Vanuatu there was a more even distribution in the sources of injury reported, with other persons, fathers, mothers and boyfriends/girlfriends being reported with similar level of frequency.

### Multivariable analysis of factors associated with intentional injury

Multivariable analysis showed that among 14–17 year olds the odds of intentional injury were higher among boys than girls in Tonga and Vanuatu. In these two populations 16 and 17 year olds were the least likely to report an injury of this type (Table 2). In Pohnpei sex or age were not independently related to intentional injury.

**Table 2:** Factors associated with intentional injury among adolescents aged 14–17 years

Variables	Intentional injury, adjusted OR (95% CI)		
	
	Pohnpei n = 1442	Tonga n = 1148	Vanuatu n = 2925
Gender			
Girl	1.00	1.00	1.00
Boy	1.15 (0.97–1.37)	1.84 (1.48–2.28)***	1.39 (1.22–1.58)***
Age			
14	1.00	1.00	1.00
15	0.81 (0.55–1.19)	0.71 (0.49–1.01)	0.80 (0.65–0.99)
16	0.87 (0.60–1.27)	0.59 (0.43–0.79)**	0.62 (0.52–0.74)***
17	0.70 (0.49–1.00)	0.53 (0.34–0.82)**	0.55 (0.44–0.69)***
Living with parent			
No	1.00	1.00	-
Yes	1.59 (1.26–1.99)**	0.69 (0.56–0.86)**	
Smoking			
<1 per week	-	1.00	1.00
1 or more per week		2.21 (1.62–3.00)***	1.52 (1.03–2.25)*
Illicit drug use			
<1 per week	1.00	-	1.00
1 or more per week	1.87 (1.55–2.26)***		1.92 (1.53–2.40)***
Have you been bullied in school this term?			
No/once/twice	1.00	1.00	1.00
Sometimes or more	1.42 (1.13–1.78)**	1.40 (1.07–1.84)*	1.66 (1.34–2.05)***
How often have you been taken part in bullying			
No/once/twice	1.00	-	-
Sometimes or more	1.73 (1.42–2.09)***		
Other students do not want to spend time with you			
None/once/twice/sometimes	-	-	1.00
Once to several times a week			1.78 (1.47–2.15)***
Easy to talk to family (mum, dad, siblings)			
Easy/very/difficult/I don't have	-	-	1.00
Very easy			1.19 (1.05–1.34)**
Easy to talk to friends/others			
Easy/very/difficult/I don't have	-	-	1.00
Very easy			1.23 (1.06–1.42)**
Teacher rating score (~quartiles):			
1 – 13 (positive)	-	1.00	1.00
14 – 17		1.00	1.46 (1.20–1.77)**
18 – 21		1.00	1.36 (1.08–1.71)*
22 – 35 (negative)		1.61 (1.28–2.03)**	1.68 (1.42–1.99)***
School rating score (~quartiles):			
1 – 13 (positive)	-	1.00	
14 – 17		1.00	-
18 – 21		1.00	
22 – 35 (negative)		2.07 (1.25–3.44)**	

n = number of observations used in final model.

All 95% CIs adjusted for clustering effect by school and corrected for finite population sampling.

Statistically significant level: * = p-value<0.05; ** = p-value<0.01; *** = p-value<0.0001. Hosmer and Lemeshow goodness-of-fit statistics for each model were: Pohnpei, χ^2^_(8) _= 9.60, p-value = 0.29; Tonga, χ^2^_(8) _= 11.85, p-value = 0.16; Vanuatu, χ^2^_(8) _= 11.09, p-value = 0.20.

Across the three societies the factor consistently associated with a higher odds of intentional injury was reporting being bullied at least sometimes in the past school term. In Pohnpei bullying other students in the past term was also associated with a greater likelihood of intentional injury, while in Vanuatu being excluded or ignored by other students at least once per week was associated with this type of injury.

There was evidence of a relationship between intentional injury and drug use. In Tonga and Vanuatu smoking at least once per week was related to a greater likelihood of this type of injury, while in Pohnpei and Vanuatu use of an illicit drug at least once per week showed a positive association with intentional injury.

Reporting a negative perception of school teachers was related to higher odds of intentional injury in Tonga and Vanuatu. In Tonga a negative overall rating of the school environment showed a similar relationship.

In Pohnpei living with parents was related to higher odds of intentional injury, whereas in Tonga the relationship was in the opposite direction. In Vanuatu reporting close relationships with family members and with friends, indicated by easy communication about personal difficulties, was related to a greater likelihood of intentional injury.

## Discussion

This study has found that at least one-third of boys and one-quarter of girls aged 14–17 years in these Pacific Island societies reported an intentional injury in the past 12 months, with a markedly higher prevalence among 11–12 year olds in the two populations where they were measured. The data highlight an adolescent health issue that has not been examined at the population level in the Pacific Islands region and which deserves closer attention by public health practitioners.

The limitations of the study need to be acknowledged before detailed consideration of the results. First, the findings may be subject to measurement bias as data were obtained by self-report and the validity and reliability of the questions has not been confirmed in the languages in which the study was conducted. All items were, however, translated and back-translated and efforts were made to improve confidentiality and accuracy by removing teachers from classrooms during the administration of surveys. Secondly, the data are only generalisable to in-school youth, whose characteristics may differ significantly from young people not in school. Thirdly, as a cross-sectional study the temporal sequence of the relationship between injury and other risk behaviours and psychosocial factors cannot be disentangled. The data do, however, highlight issues that are relevant to the problem of intentional injury that may assist in the design of injury control programs.

Despite its limitations, this is the first population based study of injury among school students in these Pacific Island societies. In the 14–17 years age group, for which comparable data were available from all locations, the highest period prevalence of intentionally inflicted injury was found in Pohnpei. A notable aspect of the finding in Pohnpei was that there was little difference between boys (62%) and girls (56%), whereas in both Tonga and Vanuatu girls were significantly less likely to have been intentionally injured than boys. The situation in these latter populations is consistent with literature, largely drawn from developed countries, which reports that boys are more likely to be intentionally injured, at least in the context of physical punishments at home [15,16], physical fighting [17,18] and bullying [19-22].

For both boys and girls in Tonga and Vanuatu, there was a decrease in intentionally inflicted injuries with increasing age. Again, this contrasted with Pohnpei, where for girls there was no apparent pattern by age and for boys levels remained stable after a peak at age 14 years. Studies concerning intentional injury among young people have reported mixed findings in relation to age trends, depending on the type of injuries examined. For example, parental mistreatment of children has been seen to increase with age in studies in the United States [15] and New Zealand [23]. Dating violence has also been shown to increase with age [24,25]. On the other hand, sibling abuse [15], physical fighting [17] and bullying [20-22] have all been found to decrease with increasing age.

For boys, the primary source of injury across all societies was 'other persons'. This category was also reported as an important source of injury for girls. Given the significant association in each of the three populations between being bullied and having sustained an intentional injury, it is likely that the 'other person' category included injuries due to violence inflicted by other students. In Pohnpei students who reported bullying others were also more likely to report an intentional injury, suggesting that bullying may have resulted in violent reprisals.

In each of the three societies boyfriends/girlfriends were reported as the second primary source of injury for boys. While this was also an important source of injury for girls across the populations, boys tended to report this more frequently. Studies conducted in other countries have reported that dating violence may be inflicted in at least equal rates between genders [24-26], indicating that it is plausible that girls in these Pacific island societies are equally as violent as boys in relationships. It is also feasible that some of the injuries sustained by boys may have been in self-defence to perceived or actual aggressive acts [26].

Parents were a frequently identified source of injury for both boys and girls across all three societies. Previous research in Pacific Island countries has found that physical punishment of children may be seen as appropriate and responsible parenting [27]. A study in the Federated States of Micronesia (FSM) found that harsh punishment for particular behaviours, including disrespect and disobedience, was acceptable provided it was restrained and did not result in serious injury [28]. In the present study it was not possible to determine whether injuries inflicted by a parent resulted from a culturally acceptable level of physical discipline, or whether they exceeded this level. Regardless of this, efforts to address this issue may be more successful if they place an emphasis on promoting adolescent social and emotional well-being, rather than directly addressing social mores regarding parenting. Framing this issue in a way that fosters dialogue among leaders and members of the wider community is a necessary step to raise awareness and develop partnerships for action.

There were inconsistent findings across the three populations about the associations between living arrangements, social relationships and intentional injury. Past studies have reported that living in an extended-family may be a protective factor for children, potentially decreasing stresses and other demands associated with child-rearing [29,30]. In support of this, residing with parents had a protective effect in terms of intentional injury in Tonga, which may be because the extended family living arrangement is common in that society [31]. In Pohnpei, on the other hand, living with parents was found to be associated with a higher likelihood of intentional injury. This may be explained by the reports of ethnographic researchers that the extended family network in FSM is breaking down, increasing psychosocial stress upon parents and children and resulting in the loss of protective mechanisms offered by multiple parenting within extended family groups [23,27]. A surprising finding in Vanuatu was that students reporting closer relationships with friends and family members were more likely to report an intentional injury. This may reflect the extent to which young adolescents are cared for by their older siblings, who could draw upon physical punishments as a way of controlling their younger sibling's behaviour because they are unfamiliar with other methods of discipline that could be used.

In Tonga and Vanuatu intentional injury was associated with smoking, while in Pohnpei and Vanuatu it was associated with illicit drug use. These findings support literature which reports that substance abuse may be correlated with violent behaviour among youth [32] and that health-risk behaviours can co-exist as part of a 'behaviour syndrome' [18]. A further issue for consideration is that the use of tobacco or illicit drugs may provoke disciplinary action which results in injury.

Several indicators of the school environment, including a negative perception of teachers in Tonga and Vanuatu and a poor overall rating of the physical and social environment at school in Tonga, were related to a greater risk of intentional injury. Research from the United States indicates that the school environment can have a significant effect upon injury risk [26], with students who believed that their teachers were supportive reported to be less likely to carry weapons, drink alcohol and engage in other delinquent behaviour, and more likely to practice health-preserving behaviours. This draws attention to the potentially important role that schools have to play in injury prevention, in developing life skills and assertiveness to improve interpersonal relationships and putting in place policies to control bullying.

## Conclusion

This study has found that intentional injury is an important issue for examination and discussion by community leaders, health and educational authorities and families in these Pacific Island societies. Action should take into account the associated issues of bullying, drug use and dissatisfaction with the school environment, with relationships between peers, boyfriends and girlfriends, and parents and children being key domains for attention. The limited availability of data about the types, severity, causes and circumstances of intentional injury needs to be addressed, not only in these Pacific Island societies but in other countries where this issue has not been thoroughly examined, in order to strengthen the evidence base for injury prevention strategies.

## Competing interests

The authors declare that they have no competing interests.

## Authors' contributions

BJS assisted with data analysis and undertook manuscript preparation. PP was responsible for study design and field work and contributed to data analysis and manuscript preparation. DB assisted with data analysis and undertook manuscript preparation. GP assisted with data analysis and manuscript preparation. MG contributed to study design and field work. DH contributed to study design and field work. TC held primary responsibility for data analysis. AB contributed to study design and data analysis. All authors read and approved the final manuscript

## Pre-publication history

The pre-publication history for this paper can be accessed here:


